# Sclerosing Mesenteritis, a Rare Cause of Mesenteric Mass in a Young Adult: A Case Report

**DOI:** 10.3389/fsurg.2021.722312

**Published:** 2021-08-20

**Authors:** Eliana Piombino, Costanza D'Agata, Maria Carolina Picardo, Claudia Caltavuturo, Gaetano Magro, Cristina Colarossi, Lorenzo Memeo

**Affiliations:** ^1^Pathology Unit, Department of Experimental Oncology, Mediterranean Institute of Oncology, Catania, Italy; ^2^Surgical Oncology Unit, Department of Experimental Oncology, Mediterranean Institute of Oncology, Catania, Italy; ^3^Radiology Unit, Department of Experimental Oncology, Mediterranean Institute of Oncology, Catania, Italy; ^4^Department of Medical and Surgical Sciences and Advanced Technologies, G.F. Ingrassia, University of Catania, Catania, Italy

**Keywords:** small bowel obstruction, abdominal mass, mesenteric mass, fibroinflammatory disease, sclerosing mesenteritis

## Abstract

Sclerosing mesenteritis (SM) is a rare fibroinflammatory disorder that involves mesenteric adipose tissue, more frequently localized in the small intestine, with an insidious clinical presentation having symptoms related to mass effect, usually resulting in bowel obstruction, mesenteric ischemia, as well as rapid weight loss. We report a case of a 23-year-old male presenting with palpable abdominal mass, mesogastric pain, and a history of rapid weight loss, who underwent exploratory laparoscopy. A hemorrhagic and gelatinous nodular tumor mass of the mesentery was identified and the surgical procedure was converted to a laparotomic approach. Histologically, the mass was composed of a proliferation of bland-looking spindle cells with slightly eosinophilic cytoplasm and elongated normochromatic nuclei with mild nuclear atypia, haphazardly set in a collagenized stroma; fat necrosis and inflammatory cells (lymphocytes, plasma-cells, and histiocytes) were also evident. The diagnosis of sclerosing mesenteritis was made. Our case emphasizes that histology remains pre-eminent for a correct diagnosis of SM, as pre-operative radiological-based diagnosis is non-specific.

## Introduction

Sclerosing mesenteritis (SM) is a rare fibroinflammatory disorder that involves mesenteric adipose tissue, more frequently localized in the small intestine (1, 2).

Although the etiopathogenesis is still to be elucidated and is based on the single cases reported in the literature, an association with abdominal trauma, surgery, autoimmune conditions such as IgG4 disease, infection, ischemia, and malignancy have been suggested (2–5).

SM has an insidious clinical presentation with non-specific symptoms related to mass effect, usually resulting in bowel obstruction, mesenteric ischemia, as well as rapid weight loss (5–8).

Based on the predominant histological component (inflammation, fat necrosis or fibrosis), a wide variety of terms, including mesenteric panniculitis (MP) (5), mesenteric lipodystrophy (ML) (9), or retractile mesenteritis (RM) (3), has been used interchangeably.

We herein report on a case of a 23-year-old male presenting with palpable abdominal mass and mesogastric pain.

## Case Study

A 23-year-old male was admitted to our institution with a history of mesogastric pain, fatigue, loss of appetite, and rapid weight loss (about 30 kg in 1 year with a BMI reduction from 25.8 to 16.2).

Abdominal examination revealed distended abdomen with a large-sized, slightly mobile, non-tender mass in the periumbilical region. Gastroscopy and colonoscopy showed unspecific findings. Medical history was negative for previous neoplastic diseases or abdominal surgery.

Laboratory tests showed blood count with hemoglobin of 12.70 g/dl, a white blood cell count of 13.920 mmc and platelet count at 606.000 mmc; CRP was 3 mg/dL while ESR was 42 mm/h.

**Figure 1:**
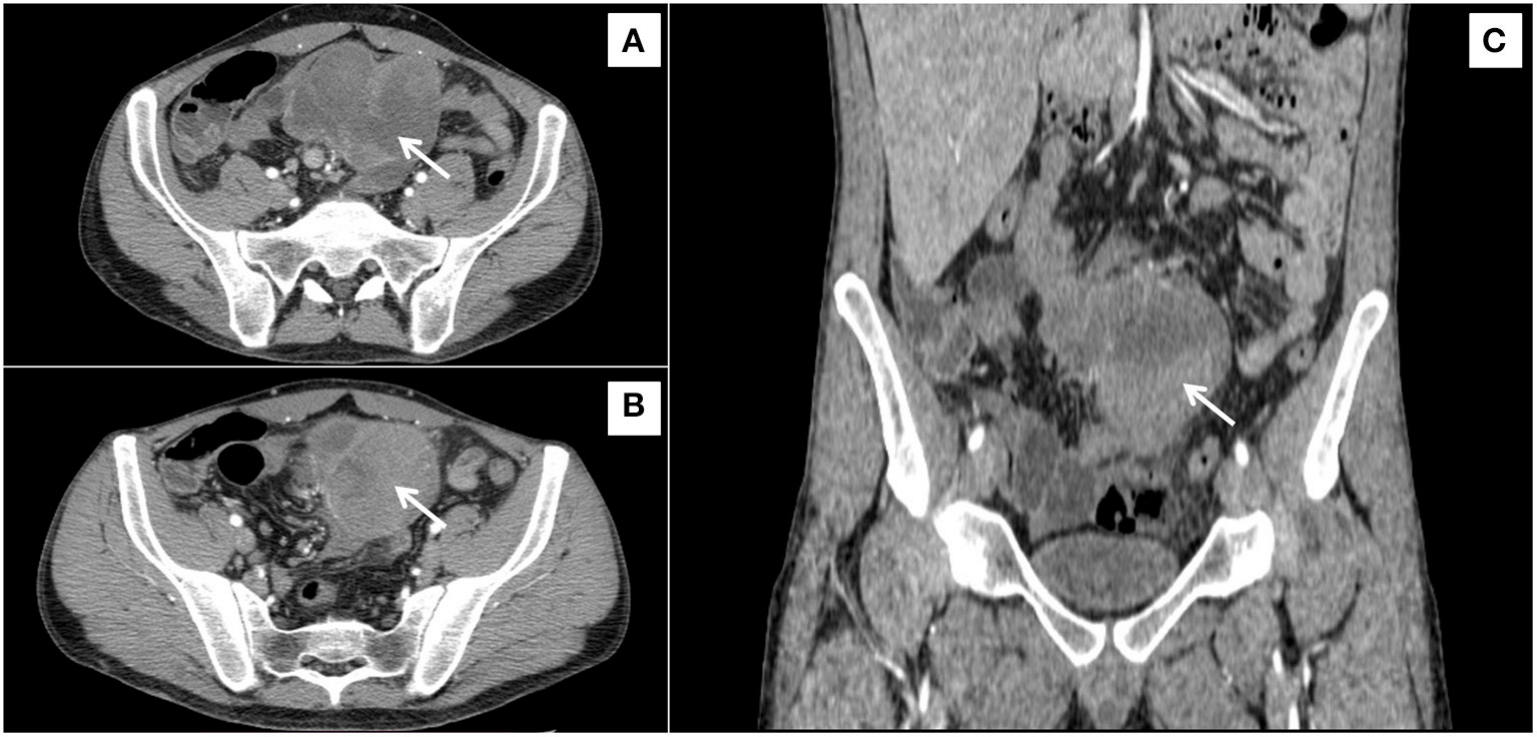
Irregular, inhomogeneous, solid soft-tissue mass, with a diameter of 90 mm, sited in the root of mesentery (arrows), in contact with the small bowel, shown from three different orientations **(A–C)**.

Given the rapid weight loss, a clinical sign indicating malignancy, a CT scan was performed. An oval-shaped mass measuring 8.5 × 8 × 6.5 cm, with evidence of a pseudo-capsule and locoregional lymph nodes of increased volume, were observed in the mesentery (Figure 1).

Therefore, an exploratory laparoscopy was planned. A large-sized, solid mass arising from the small bowel mesentery, with multiple hemorrhagic spots, was seen; the wall of the small intestine appeared thinned and extensive intraperitoneal adhesions were present. Laparotomic excision of the mass, with small bowel resection and primary anastomosis were performed. Post-surgical days were uneventful (Class 0 on the Clavien-Dindo Classification) and patient was discharged on the sixth day post-intervention.

**Figure 2:**
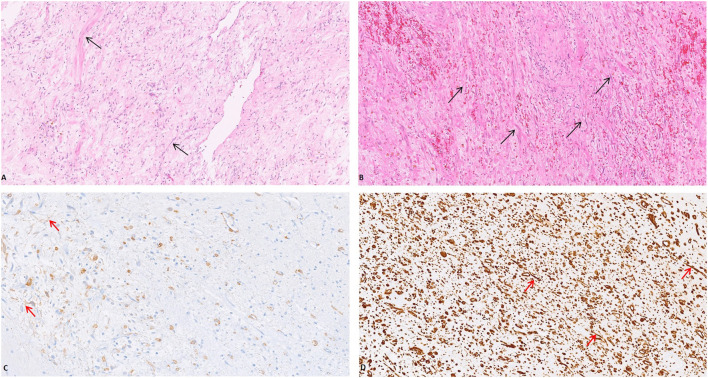
The mesenteric mass was composed of a proliferation of bland-looking spindle cells with slightly eosinophilic cytoplasm and elongated normochromatic nuclei with mild nuclear atypia, haphazardly set in a collagenized stroma [**(A,B)** (Arrow), H&E, 20x]. The myofibroblastic nature of the lesional spindle-shaped cells was confirmed by the expression of Smooth Muscle Actin [**(C)** (Arrow), 20x] and Vimentin [**(D)** (Arrow), 20x].

Grossly, a hemorrhagic and gelatinous nodular tumor mass of the mesentery, measuring 8.5 cm in its greatest dimension, was identified. Histologically, it was composed of a proliferation of bland-looking spindle cells with slightly eosinophilic cytoplasm and elongated normochromatic nuclei with mild nuclear atypia, haphazardly set in a collagenized stroma; fat necrosis and inflammatory cells (lymphocytes, plasma-cells, and histiocytes) were also evident. Mitoses, including atypical forms, and nuclear pleomorphism were absent. Muscolaris propria and subserosal of the small intestine showed vascular congestion, while the mucosa of the small intestine was unremarkable. Immunohistochemical analyses, showing a diffuse staining for vimentin and smooth muscle actin, revealed the myofibroblastic nature of the lesional spindle-shaped cells (Figure 2). Desmin, CD117, CD34, DOG1, β-catenin, S100 protein, pancytokeratins, and EMA were negative. The plasma cell component was negative for IgG4. The patient was discharged on day 6 after surgery with a diagnosis of sclerosing mesenteritis. No predisposing condition (trauma, previous surgery, infection, auto-immune disease, neoplasms, or ischemia) was found. After 10 months of follow-up, the patient was in good condition and CT scan was negative.

## Discussion

SM is a rare fibroinflammatory disorder that involves mesenteric adipose tissue, more frequently localized in the small intestine. Rarely, it may be localized in the retroperitoneum, the peri-pancreatic region or the pelvis (6, 10).

SM was first described as “*retractile mesenteritis*” by Jura et al. in 1924 (11) and over the years, several names have been proposed including RM (11), MP (5), and ML (9). Nowadays, the unifying term “SM” is the most used in the literature.

The epidemiology of SM is not well-defined; autopsy studies by Kuhrmeier estimate an incidence of 1% (9/712 autopsies) (12). There are also several radiological studies that estimate an incidence of 0.6% but lacking histological confirmation (2, 4, 8). SM typically occurs in the fifth/sixth decade of life, but occasional cases have also been documented in pediatric age. Most studies show a male prevalence with a Male/Female ratio of 2:1 (3, 4).

The etiology of SM remains unknown; the etiopathogenetic hypotheses derive from cases reported in the literature and include association with trauma, surgery (5, 11), autoimmune disease (4), neoplasm (5, 7), infection (13), and ischemia (14). The iatrogenic and the traumatic etiology can be explained as a disproportionate response to the surgical wound (2). The onset of SM is often associated with autoimmune conditions, such as IgG4 mediated sclerosing disorders, Lupus, rheumatoid arthritis, Riedel thyroiditis, and primary sclerosing cholangitis (15, 16). The role of autoimmunity in SM is also supported by the evidence of response to immunomodulatory therapies (4, 15, 16). In our case no predisposing factor was noted nor were there history of previous surgery, trauma, autoimmune disease, infection, or neoplasms.

The association between SM and malignancies still remains a matter of debate; in favor of the association are the studies of Ogden et al. (5) and of Jura (11), which demonstrated an association with lymphoma in 8 out of 53 and 2 out of 7 patients, respectively. In the cohort of patients studied by Kipfer, 16 out of 53 patients also presented malignant neoplasms. Against these hypotheses are, instead, the studies of Gogebakan et al. (17) that showed no statistical difference between patients with sclerosing mesenteritis with underlying malignancy and a control group.

Symptoms are highly non-specific and include: abdominal pain, weight loss, presence of palpable mass, as shown by our patient, as well as fever, nausea and vomiting (6). Our patient experienced non-specific symptoms only, with only a few months of mesogastric pain and a 30 Kg weight loss in 1 year.

There are no specific laboratory tests for SM; in most of these patients there is an increase in inflammation indices, such as erythrocyte sedimentation (ESR) rate and c-reactive protein (CRP) (18); values of both ESR and CRP were increased in our patient.

The advent of the CT scan has improved radiological diagnostics of SM, although the differential diagnosis with malignancy can be very difficult, especially when locoregional lymphadenopathies are present, as in our case. Signs of specificity are represented by the “fat ring sign” and by the presence of a pseudocapsule (5, 18–20).

Coulier proposed criteria for diagnosing SM: a mesenteric mass (1) causing a mass effect without invasion of surrounding structures, (2) high attenuation [250–270 Hounse field units (HU) vs. 2,100–2,120 HU for subcutaneous fat], (3) mesenteric fat containing small (10 mm) soft tissue nodes with (4) hypo-attenuating fat surrounding the lymph nodes or mesenteric vessels (“fat halo sign”) and (5) an over-attenuating pseudocapsule surrounding the absence of ascites or known neoplasm involving the mesentery (21). These criteria have not been validated and their usefulness in differential diagnosis is a matter of debate.

Despite the improvement in imaging methods, the histological evaluation of biopsy and surgical samples remains the gold standard for the diagnosis of SM. Grossly it may present as a single mass, multiple nodules, or as a diffuse mesenteric thickening (1).

Histologically it is characterized by a variable admixture of fibrosis, chronic inflammation and fat necrosis.

Kipfer proposed a classification of SM into three subgroups. Type I SM (diffuse mesenteric thickening): thickened mesentery up to 10 cm. The thickening typically ended within 3–5 cm of the mesenteric border. Type II SM (single discrete tumor): often localized in the jejunal mesentery. The mass can be smooth or multi-lobular, firm, or rubbery consistency. Type III SM: multiple nodules with the same consistency and features as describes in type II (7). Our patient was classified as a type II SM since a single, discrete 8.5 cm mass was seen in the mesentery.

Some authors have emphasized the link between SM and IgG4 disease, by showing the presence of IgG4 positive plasma cells in the context of SM; histologically, in these cases there is also the presence of storiform fibrosis and obliterative phlebitis (6, 22, 23). The absence in these patients of the other criteria of IgG4 diseases, such as multi-organ involvement or the elevation of serum IgG4 levels, did not clarify the clinical significance of this pathological finding. In the present case, the plasma cell component was negative for IgG4.

The main differential diagnoses of SM revolve around GIST (gastrointestinal stromal tumor) and mesenteric fibromatosis. GIST, the most common mesenchymal tumor of the gastrointestinal tract, is more cellular than SM, and it is characteristically stained with CD117, CD34, and DOG-1, markers that were negative in our case. Unlike desmoid-type fibromatosis, SM lacks a fascicular arrangement with parallel aligned cells, as well as the expression of β-catenin (24, 25). Malignant mesenchymal tumors were ruled out based on the absence of severe nuclear atypia, high mitotic index and necrosis.

No consensus has been reached regarding the management of SM; as far as medical treatments are concerned, the use of corticosteroids, tamoxifen, thalidomide, colchicine, infliximab, azathioprine, and cyclophosphamide has been proposed with variable results (26). In cases where obstructive symptoms prevail, as in our patient, surgical excision is mandatory.

The prognosis of SM is generally excellent, with the painful symptoms subsiding and the mass reducing in size in most patients (1, 3). For patients non-surgically treated, some authors have reported an improvement in inflammatory markers such as ESR and CRP with successful treatment; however, this is not universal and these markers may not even be elevated on presentation (1), while monitoring of surgically treated patients is usually limited to periodic CT scans (1).

## Conclusions

Our case emphasizes that histology remains prominent for a correct diagnosis of SM, as preoperative radiological-based diagnosis is non-specific. When the radiologist is faced with a mesentery mass of the small bowel, SM should be included in the differential diagnosis, in view of its benign clinical behavior and different management, compared to the most common neoplasms arising in the same site.

## Data Availability Statement

The original contributions presented in the study are included in the article/supplementary material, further inquiries can be directed to the corresponding author/s.

## Ethics Statement

Ethical review and approval was not required for the study on human participants in accordance with the local legislation and institutional requirements. The patients/participants provided their written informed consent to participate in this study.

## Author Contributions

EP drafted the manuscript. CD'A, MP, and CCa provided the clinical information of the patient. GM, CCo, and LM edited the manuscript. All authors were involved in the clinical care of the patient and approved the final version of the manuscript at the time of submission.

## Conflict of Interest

The authors declare that the research was conducted in the absence of any commercial or financial relationships that could be construed as a potential conflict of interest.

## Publisher's Note

All claims expressed in this article are solely those of the authors and do not necessarily represent those of their affiliated organizations, or those of the publisher, the editors and the reviewers. Any product that may be evaluated in this article, or claim that may be made by its manufacturer, is not guaranteed or endorsed by the publisher.
